# Executing SADI services in Galaxy

**DOI:** 10.1186/2041-1480-5-42

**Published:** 2014-09-22

**Authors:** Mikel Egaña Aranguren, Alejandro Rodríguez González, Mark D Wilkinson

**Affiliations:** Biological Informatics, Centre for Plant Biotechnology and Genomics (CBGP), Technical University of Madrid (UPM), Campus of Montegancedo, 28223 Pozuelo de Alarcón, Spain; Genomic Resources, Department of Genetics, Physical Anthropology and Animal Physiology, Faculty of Science and Technology, University of Basque Country (UPV/EHU), Sarriena auzoa z/g, 48940 Leioa - Bilbo, Spain

**Keywords:** Galaxy, Web services, SADI, RDF, SPARQL, OWL

## Abstract

**Background:**

In recent years Galaxy has become a popular workflow management system in bioinformatics, due to its ease of installation, use and extension. The availability of Semantic Web-oriented tools in Galaxy, however, is limited. This is also the case for Semantic Web Services such as those provided by the SADI project, *i.e.* services that consume and produce RDF. Here we present SADI-Galaxy, a tool generator that deploys selected SADI Services as typical Galaxy tools.

**Results:**

SADI-Galaxy is a Galaxy tool generator: through SADI-Galaxy, any SADI-compliant service becomes a Galaxy tool that can participate in other out-standing features of Galaxy such as data storage, history, workflow creation, and publication. Galaxy can also be used to execute and combine SADI services as it does with other Galaxy tools. Finally, we have semi-automated the packing and unpacking of data into RDF such that other Galaxy tools can easily be combined with SADI services, plugging the rich SADI Semantic Web Service environment into the popular Galaxy ecosystem.

**Conclusions:**

SADI-Galaxy bridges the gap between Galaxy, an easy to use but “static” workflow system with a wide user-base, and SADI, a sophisticated, semantic, discovery-based framework for Web Services, thus benefiting both user communities.

## Background

There is a growing global movement towards representation of bioinformatics data and knowledge using contemporary syntaxes and semantic languages approved by the World Wide Web Consortium (W3C) [1], like Resource Description Framework (RDF) [2] and Web Ontology Language (OWL) [3]. Major bioinformatics resources making their data available using these formats include UniProt [4], EBI [5], and soon, the DNA Databank of Japan [6]. Beyond these core providers, there are also large integrated warehouses of bioinformatics data in RDF format including, most significantly, Bio2RDF [7], which integrates critical bioinformatics resources such as dbSNP [8], OMIM [9], and KEGG [10], and NCBI eutils [11], which wraps NCBI databases as resolvable RDF resources.

This wealth of resources brings the inevitable requirement for tools that support the flow of native RDF data through a formal bioinformatics analysis pipeline. The Semantic Automated Discovery and Integration (SADI) project has established design-patterns for bioinformatics resources that wish to natively consume and produce RDF data [12], and there are SADI plug-ins to several popular data workflow and exploration environments, including Taverna [13] and the IO Informatics Sentient Knowledge Explorer [14]. While Taverna is a rich and full-featured environment for constructing and editing complex workflows, Galaxy [15] is showing itself to be a favorite of bench-biologists due to its relative simplicity compared to Taverna, and the “familiarity” it brings biologists by exposing the tools they commonly use in a manner that they can quickly interpret and work with. As such, it was desirable to bring support for SADI-based, RDF-native data and analysis tools into the Galaxy environment. Here we describe SADI-Galaxy - a set of tools that retrieve and “wrap” SADI Semantic Web Services in a manner that allows them to be included in Galaxy workflows.

## Implementation

**Figure 1:**
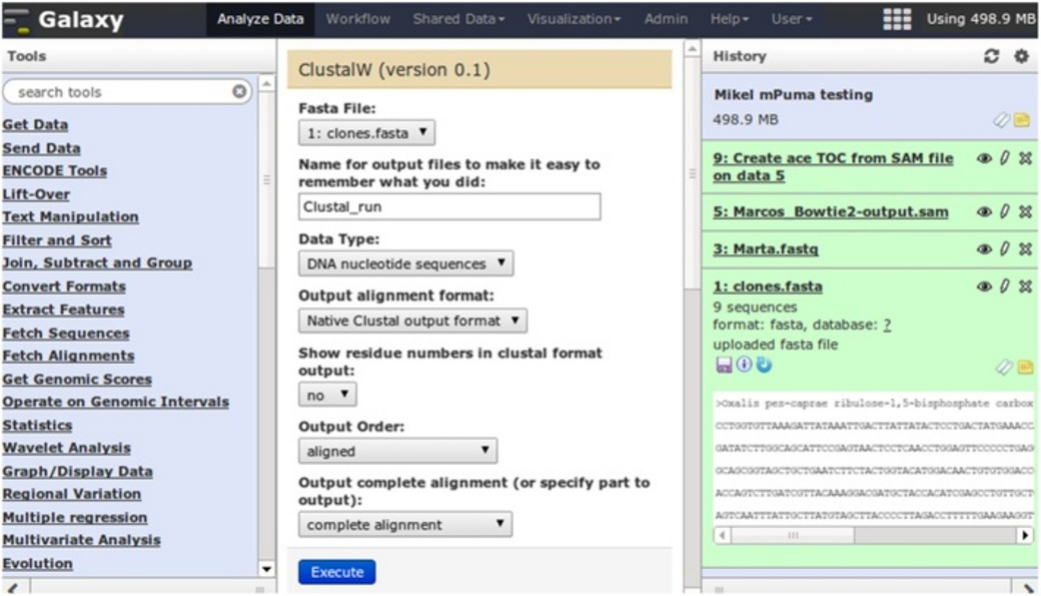
**Galaxy interface.** The Galaxy web interface is divided in different views, listed at the top: “Analyze data”, “Workflow”, “Shared Data”, “Visualization”, “Admin”, “Help”, and “User”. The frontpage is the “Analyze data” view, shown in this figure, with three columns: available tools (left), current tool (center), and history of loaded data and executed tools (right). In this example the logged in user is working in a history named “Mikel mPuma testing”: different datasets have been loaded (Steps 1, 3, 5) and a tool has been executed (The execution of tool Create ace TOC from SAM, using the result of step 5 as input, has resulted in the dataset stored in step 9). The tool ClustalW is selected to be executed next, using the dataset of step 1 as input, and the result will appear in the history as step 10. (Some steps have been deleted from the history).

Galaxy is a Web server, written in Python, that offers a very usable interface for the typical bioinformatics computational analyses (Figure 1). A user can store data, and analyse it using a variety of tools, sending the output of one tool as input to the next. This process is stored in a chronological history from which workflows can be extracted, providing an easy-to-reproduce abstraction of common steps. Data, histories and workflows are Web-shareable and can be imported and exported. A Galaxy tool is, typically, a wrapper for a terminal executable program. Since such wrappers are defined by an XML file describing the inputs and outputs of the tool as well as its Web interface [16], creating Galaxy tools from pre-existing executables is not technically demanding.

SADI is a standards-compliant set of lightweight design patterns for publishing bioinformatics data and analysis services on the Semantic Web. It uses Semantic Web technologies at every level of the Web services “stack”. In particular, a SADI service describes its interface in OWL, and then both consumes and produces RDF data that match that OWL logical description. Finally, SADI requires that the output data is semantically connected to the input data by a meaningful relationship. As such, workflows of SADI services output unbroken chains of RDF Linked Data [17]. SADI services are catalogued in a publicly-accessible database (registry), and queries against that database will be used in this study to find, and retrieve the interface definitions for, SADI services of interest to any given Galaxy user; the retrieved service definitions will become templates for the Galaxy “wrapper”, and thus the services can be accessed through these “wrappers”.

**Figure 2:**
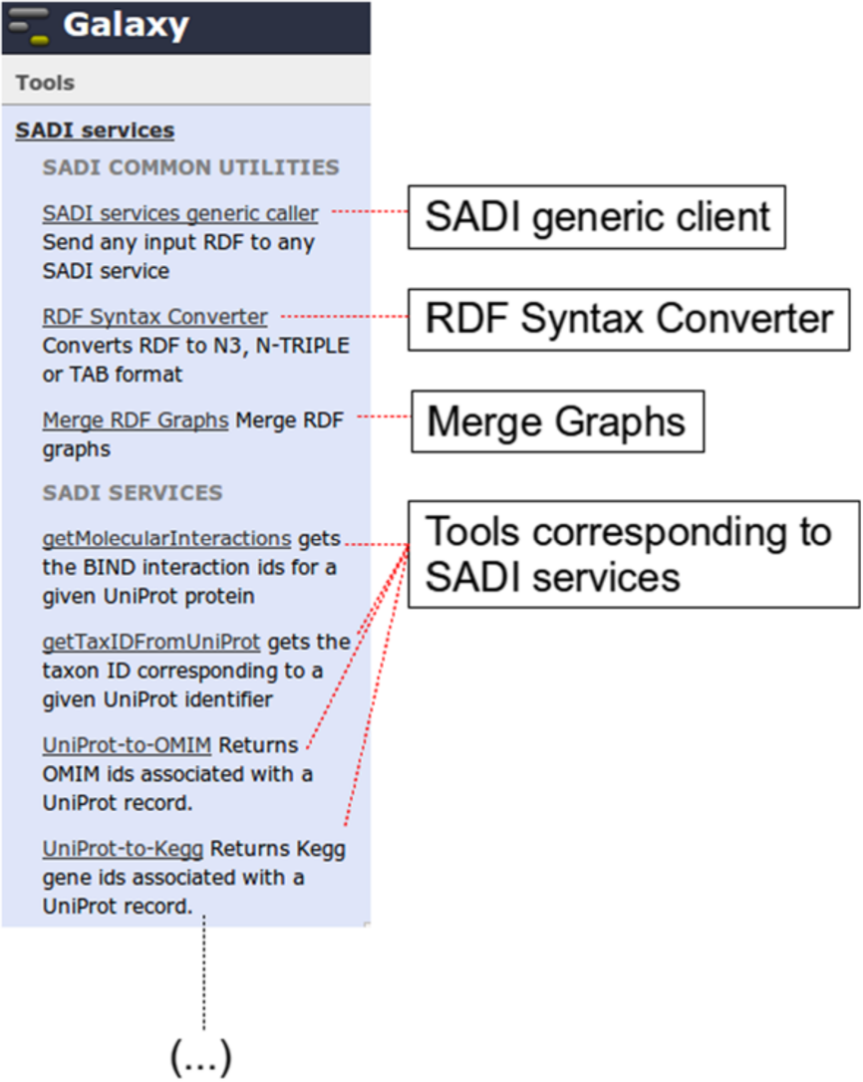
**SADI-Galaxy Core tools and SADI services as Galaxy tools.** Galaxy “Analyze data” view (only left column shown) resulting from the installation of the SADI-Galaxy Core (SADI generic client, RDF Syntax Converter, and Merge RDF Graphs, under “SADI COMMON UTILITIES”) and a number of specific SADI services, retrieved by a SPARQL query through the SADI-Galaxy Tool-generator (Under “SADI SERVICES”; only a few shown).

SADI-Galaxy consists of two parts (Figure 2): the “Core”, and the Tool-generator. SADI-Galaxy Core includes three Galaxy tools that are installed in the same manner as any other Galaxy tool [18], and they can be used on their own. These tools are:

-  
SADI generic client. This tool is able to execute any SADI service, given the service’s URI and an RDF input file that is compatible with the service. The RDF output of the service is stored as any Galaxy output. Automated reasoning is used to check if the RDF input is compliant with the SADI Service’s input OWL class; *i.e.* whether the RDF instance is inferred to be a member of the input OWL Class, effectively providing up-front, “low-cost” automated workflow validation.

-  
RDF Syntax Converter. This tool is able to convert an RDF file into a variety of formats, most importantly including Tab Separated Values (TSV) format (three columns for subject, predicate and object), so that non-RDF-based Galaxy tools can consume SADI’s output.
-  
Graph Merge. This tool is able to merge the output of different SADI services into a single RDF graph for downstream processing.

**Figure 3:**
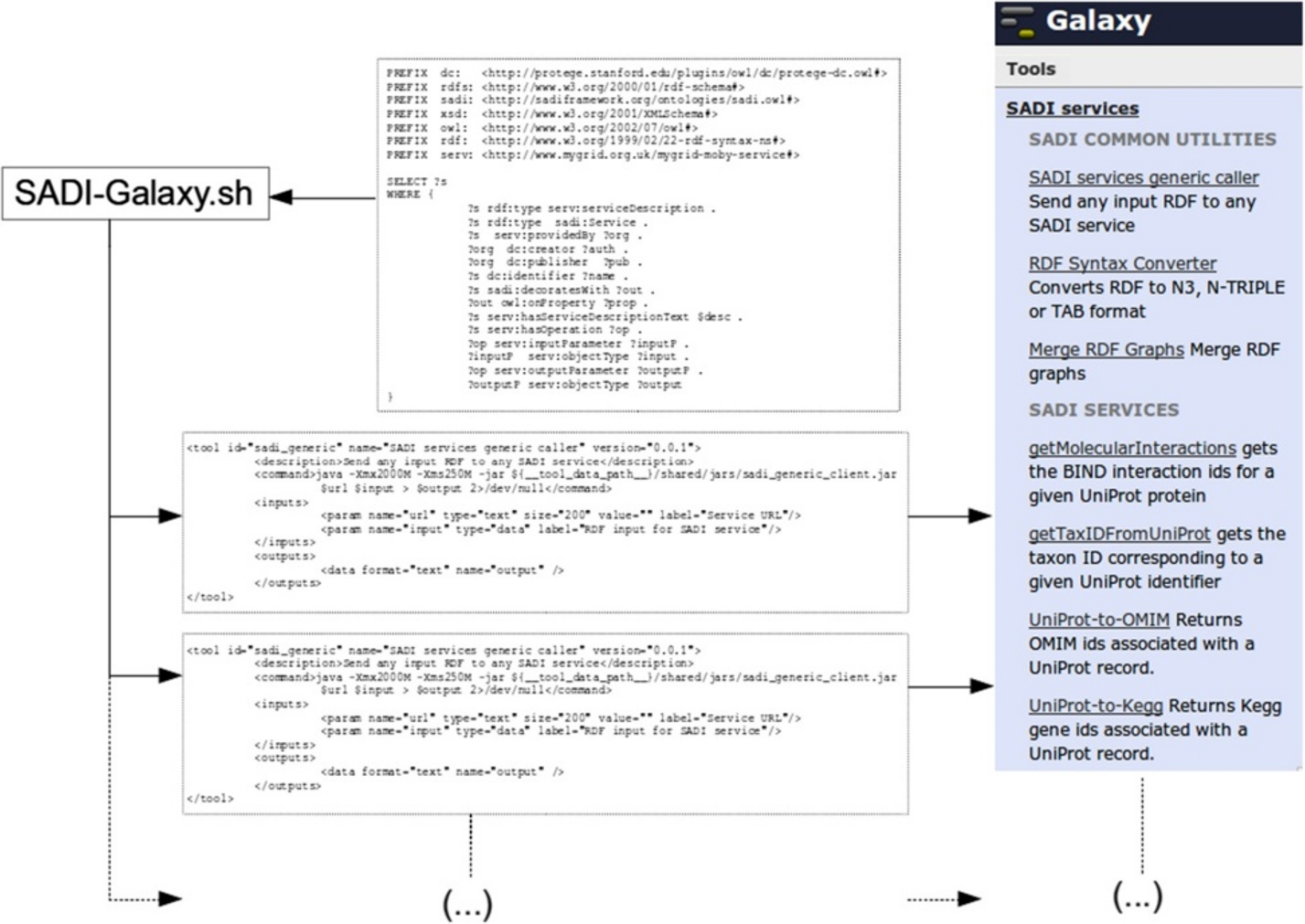
**SADI-Galaxy Tool-generator.** The Tool-generator is a Shell script that reads a SPARQL query (top, center) and generates XML files (an XML file for each SADI service Galaxy Tool). The XML files are copied to the appropriate location on the Galaxy server and appear as regular tools in the Galaxy tools menu, under “SADI SERVICES”.

SADI-Galaxy then offers an additional, more advanced functionality through the Tool-generator (Figure 3). The Tool-generator adds the ability to query a SADI Service registry, using arbitrary query parameters, to retrieve a set of matching SADI services. These Service-specific tools will then be deployed in Galaxy, alongside the Core tools described above. The Tool-generator, at the code-level, is simply a command line executable (A Shell script) that reads one or more SPARQL [19] queries and executes them against a SADI registry. The matching service URIs retrieved by the query are then used to generate, for each service, a Galaxy compliant wrapper and install it as a new Tool.

**Figure 4:**
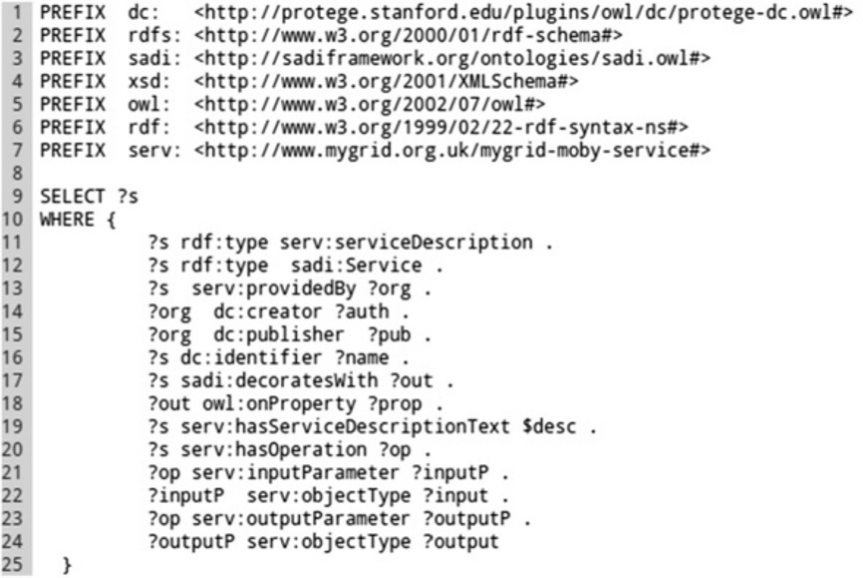
**Tool-generator default SPARQL query.** In this query a set of filters for retrieving SADI service URIs is defined: the service (?s) must be a member of the classes serv:serviceDescription and sadi:Service (Lines 11 and 12); the service must be provided by an organisation (serv:providedBy ?org, line 13); the service must add an OWL property predicate to the output (sadi:decoratesWith ?out. ?out owl:onProperty ?prop, lines 17 and 18); *etc.* The query returns around 250 active SADI services from the default registry. Other queries can be defined, as long as the ?s variable is used for service URIs. For example, in line 15, the ~wilkinsonlab.info~ value can be used, instead of the variable ?pub, to retrieve SADI services provided by wilkinsonlab.info.

The SPARQL queries used by the Tool-generator can be tuned in order to install a concrete set of services matching, for example, a particular research objective^a^. Query examples are provided in the SADI-Galaxy bundle, as well as a query generator that is able to produce parameterised queries from a base query (*e.g.* for different SADI service publishers). The default query can be seen in Figure 4.

The Galaxy tools created by the Tool-generator utilize the Core’s generic SADI client, but pre-configure many of the parameters of that generic tool. Therefore, rather than requiring users to know, for example, the address and data-types of a service, each is provided as an independent Galaxy tool, enabling simple invoking and storing of desired SADI tools as “bookmarks”. These “bookmarks” also enable Galaxy’s tool-search function to explore SADI Service descriptions. In this way, the same SADI Service can be invoked either through the generic client (if the URI is known by the user) or through the corresponding Galaxy tool (if the URI is unknown and/or the service is used frequently).

## Results^b^

### A simple example

**Figure 5:**
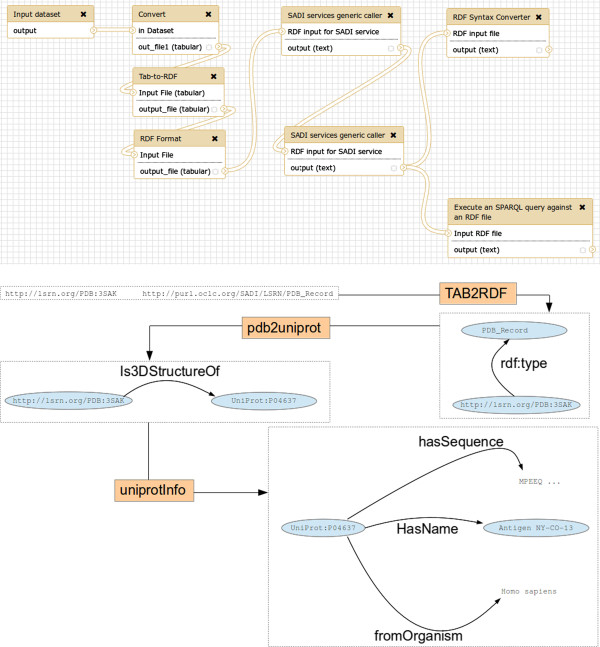
**Galaxy workflow for use case “A simple example”.** Top: Galaxy “Workflow view” interface; bottom: simplified version, with detailed depiction of files, including RDF triples (not all the triples shown). The workflow starts with a TSV file containing the information that will be sent as input to the SADI service pdb2uniprot (Input dataset). The file is processed to convert it to a column format Galaxy can recognise (Convert) and then transformed to RDF with the Tab-to-RDF and RDF format tools from the SPARQL tools tool-set. The RDF file is submitted to the pdb2uniprot SADI service using the SADI services generic caller and the output RDF is sent to the uniprotInfo SADI service, also with the SADI services generic caller. The output RDF from the uniprotInfo SADI service can be converted to a TSV file with RDF Syntax Converter or queried with SPARQL (Execute an SPARQL query against an RDF file) to obtain concrete information (Also in TSV format). Note that triples are added to the RDF input as a result of executing a SADI service, in pdb2uniprot and uniprotInfo.

To demonstrate how SADI-Galaxy can be used to transform data to RDF, in order for SADI services to consume it, and how different SADI services can be combined as regular Galaxy tools, a simple use case was envisioned, and captured in the workflow depicted in Figure 5. The aim of the workflow is to obtain the UniProt entry associated with a PDB entry [20], that is, to obtain information of the protein whose 3D structure is described in PDB. The workflow starts from a TSV file (as it is customary in Galaxy) and the TAB2RDF tool^c^, part of the SPARQL tools tool-set [21], is used to transform it to the RDF/XML syntax that a SADI service can consume. The file is submitted to the SADI service pdb2uniprot to obtain the UniProt ID, which is returned as an RDF file. The RDF file is submitted to another SADI service, uniprotInfo, to obtain all the information about that UniProt entry, also as an RDF file. This final RDF file can be converted to a TSV file with RDF2TAB, or queried with SPARQL-Galaxy to obtain concrete information about the protein [22].

### Galaxy as a complex SADI client

**Figure 6:**
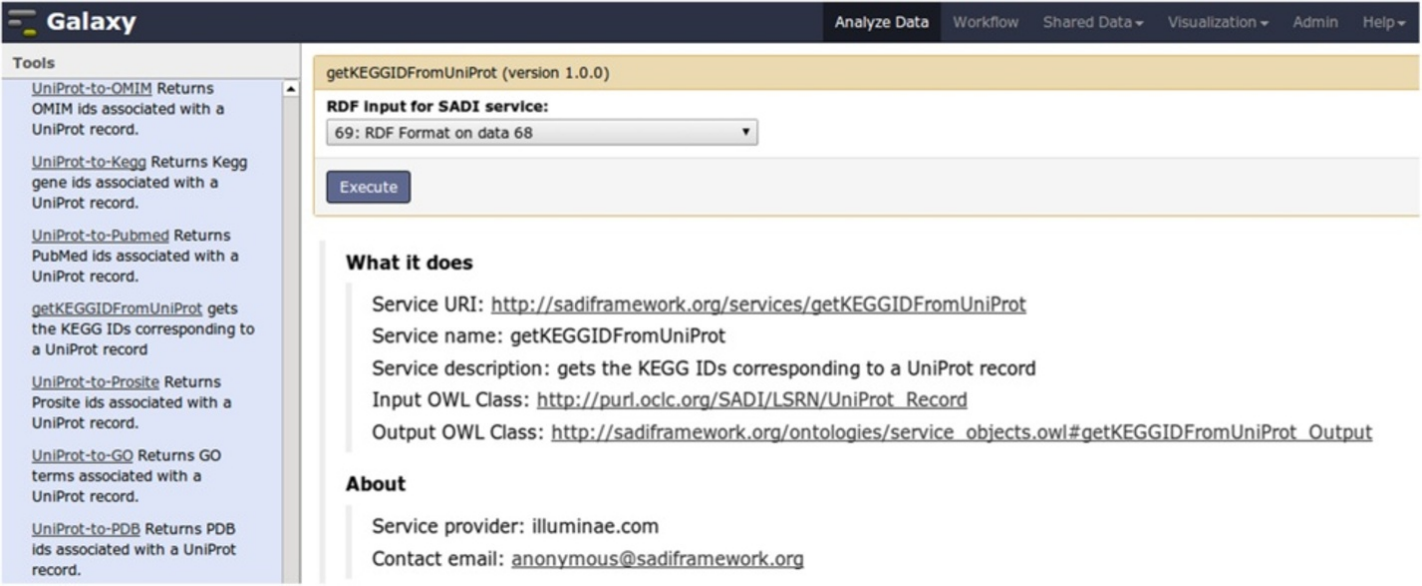
**SPARQL query to retrieve SADI services for** UniProt_Record **.** This query, when executed by SADI-Galaxy, retrieves the URIs of all the services that have UniProt_Record as input class (Lines 10 to 12): the services consume RDF data containing instances that are inferred to be members of UniProt_Record when automated reasoning is applied, *i.e.* instances that satisfy the restrictions defined in the OWL Class UniProt_Record.

**Figure 7:**
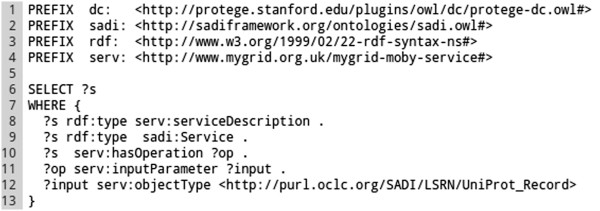
**Galaxy interface for** getKEGGIDFromUniProt **SADI service.** On the left column, available SADI services that comply with UniProt_Record are listed. On the right column, one of them, getKEGGIDFromUniProt, is selected in order to invoke it: only the RDF data must be provided (In this case, the RDF is produced by RDF format in step 69 of current history). When the “Execute” button is clicked, automated reasoning will be used to validate the RDF data against the UniProt_Record OWL Class, as noted by the “Input OWL Class” menu item in the “What it does” section of the tool interface.

**Figure 8:**
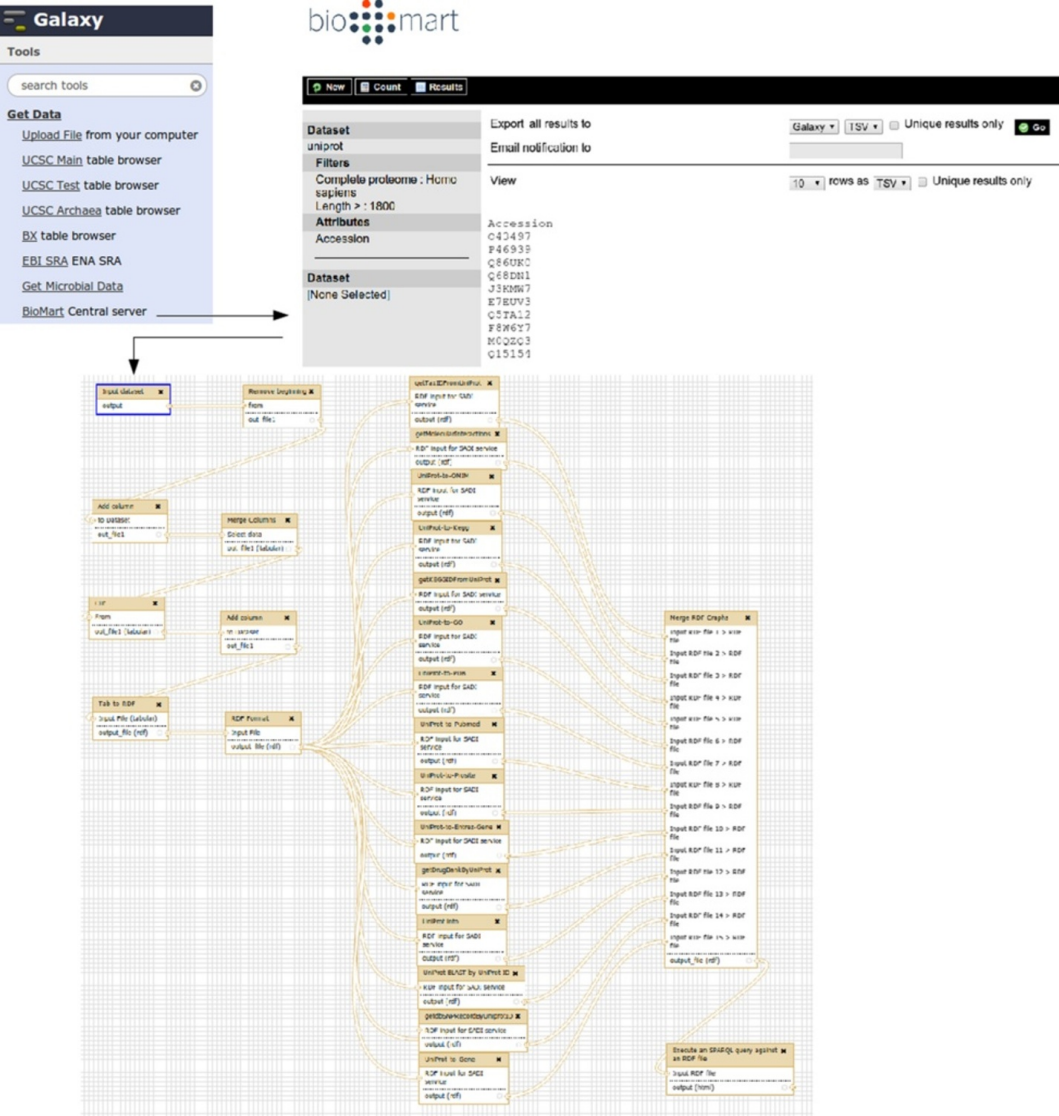
**Galaxy workflow for use case “Galaxy as a complex SADI client”.** In order to obtain the input for the workflow (Bottom), data is imported directly from a BioMart server, without having to upload the data to the Galaxy server (Top). The data, a list of UniProt IDs, is then converted to suitable RDF (Compliant with UniProt_Record) using the standard Galaxy text manipulation tools (Bottom workflow, left-hand). The resulting RDF is then sent to different SADI services, generated by SADI-Galaxy, that can consume it (Bottom workflow, middle); each service is executed as shown in Figure 7. The output of the services is merged with the Merge Graphs tool (Bottom workflow, right-hand), and queried with SPARQL Galaxy (Executed as shown in Figure 9) to generate the results shown in Figure 10.

**Figure 9:**
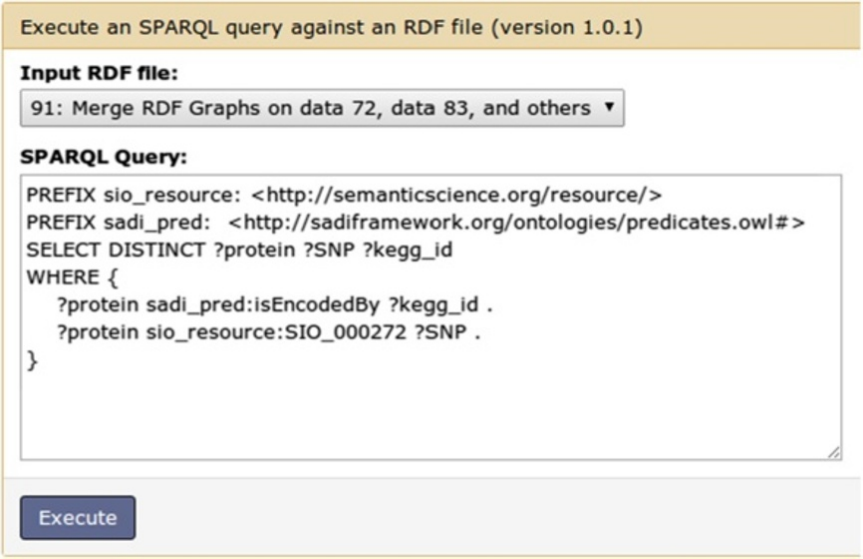
**SPARQL Galaxy.** This query takes as input the merged output of all the SADI services and finds the proteins that are encoded by a KEGG gene and are related to SNPs, to produce the results shown in Figure 10.

**Figure 10:**
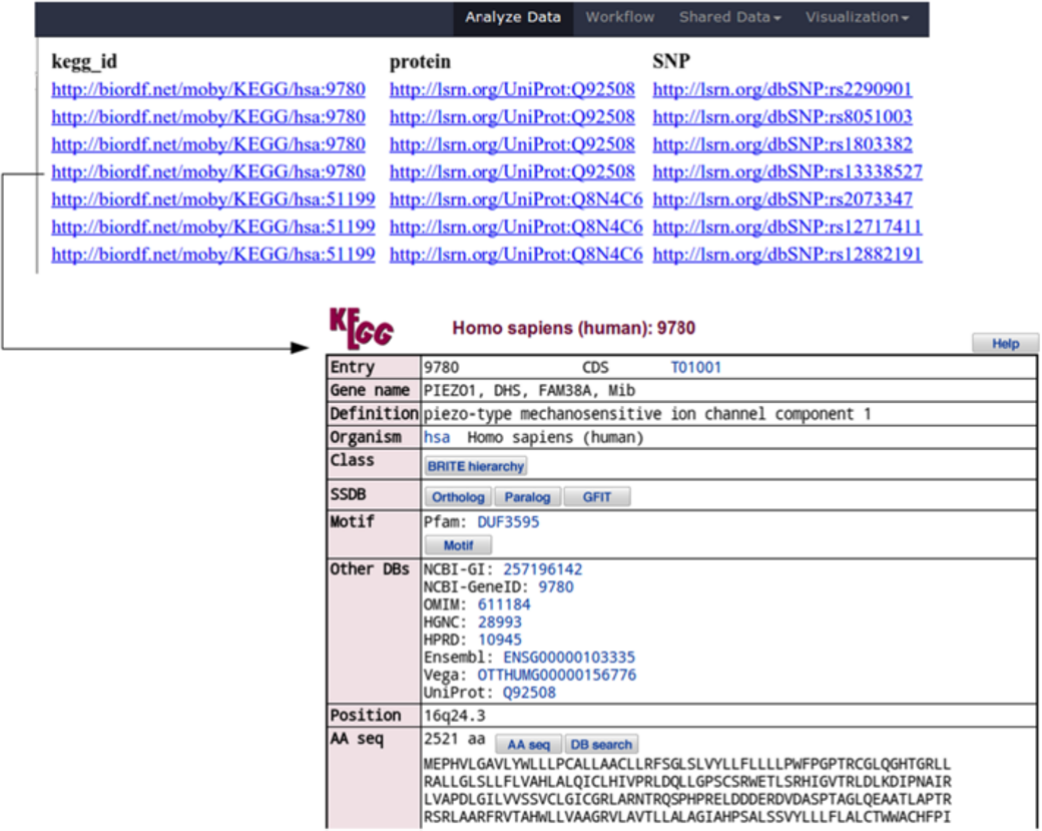
**SPARQL query results.** Galaxy interface for HTML results of query from Figure 9 (Top). The links point to actual resources: this is shown by the resolved KEGG gene entry (Bottom), obtained as the result of clicking on a link.

This second use case will reveal the more detailed functionality provided by SADI-Galaxy, including registry searching, reasoning, and RDF format conversions that allow linking between SADI tools and other Galaxy tools. The premise of this use-case is that a researcher is interested in retrieving all of the information about a specific protein, that can be obtained from any SADI service, and then to integrate and query it, as shown in Figures 6, 7, 8, 9, 10. The first step is to generate Galaxy Tools for all services that can consume data that complies with (*i.e.* is logically inferred to be a member of) the OWL class UniProt_Record [23]. This is accomplished by using SADI-Galaxy to execute the SPARQL query shown in Figure 6. As a result, the researcher obtains a Galaxy interface in which only the SADI services relevant to their investigation are presented, all pre-configured and ready to invoke their individual SADI services as shown in Figure 7. The researcher’s starting data is first retrieved from the Biomart central server [24] using the Galaxy standard facility “Get data: Biomart central server” (Top of Figure 8). The input - a list of UniProt IDs -, is manipulated with the Galaxy default text manipulation tools to generate an RDF file the SADI services can consume (Left-hand of Galaxy workflow in Figure 8). An interface to execute each SADI service is made available to the user by simply clicking on the service’s name (No need to know the service’s URI, as shown in Figure 7). Since the outputs from all SADI services (Center of Galaxy workflow in Figure 8) are all RDF, merging them is trivial (Right-hand of Galaxy workflow in Figure 8) and results in an integrated dataset that can be queried (Figure 9) to obtain results that, in turn, resolve to actual resources on the Web (Figure 10).

This process, in which the user integrates disparate information produced by many different SADI services, can be executed any time new UniProt IDs are obtained, or any time new SADI services that can consume UniProt entries are published in the registry (This can be accomplished by simply running the Tool generator with the same query periodically, without having to know when the registry is updated, *i.e.* which new services have been added).

## Discussion

SADI-Galaxy is inspired by the Galaxy Web Services Extensions (GWSE) tool [25, 26], which is able to dynamically load SAWSDL/WSDL web services as Galaxy tools. SADI-Galaxy focuses on SADI’s Semantic Web-compliant services, rather than SAWSDL/WSDL services, and therefore for the first time brings Semantic Web resources into the native Galaxy environment. In addition, the two extensions have slightly different behaviors. Where the WS Extensions tool dynamically loads new Galaxy tools, the ability to execute such dynamic loading is not part of the standard Galaxy distribution. The SADI-Galaxy code conforms strictly to the Galaxy specification, thus works in any Galaxy off-the-shelf installation. After a restart, the SADI-Galaxy generic client becomes available in the left-hand column of the Galaxy interface, and individual SADI services may be dynamically loaded into that tool by providing the service URI. To simplify this task even further, SADI-Galaxy allows the option of querying the SADI service registry to discover the URIs of desired services, thus making it possible to automatically generate a large number of desirable Galaxy tools, a powerful feature that is not available within the WS Extensions tool.

Another system that has features comparable to SADI-Galaxy is Tavaxy [27], which is a standalone server that is a “mediator” between Galaxy and Taverna. Where Tavaxy makes it possible to mix Taverna and Galaxy workflows, SADI-Galaxy concentrates on bringing Semantic Web Services - already available within Taverna - into the Galaxy environment. A similar result could be achieved by first building a workflow of SADI services in Taverna, then importing that workflow into Tavaxy in order to add the Galaxy services; however, that process is far from seamless. It is more desirable to provide native access to the thousands of SADI resources from within the Galaxy environment itself, than to require Galaxy users to use “foreign” tools such as Taverna and Tavaxy.

We suggest SADI-Galaxy as the “minimum” infrastructure that marks the point of intersection between SADI Services and Galaxy, which can now act as the core codebase upon which more powerful functionality is constructed. In particular, we expect two major developments in the near future: discovery, where given an RDF input, Galaxy is able to infer and automatically select the appropriate SADI Tool; and adding tools dynamically (as WS Extensions already does), once a consensus has been reached by Galaxy developers on how to implement a standard function for dynamic tool loading.

## Conclusions

The simplicity and predictability of the SADI Service design patterns - effectively, to simply consume and produce raw RDF over HTTP POST - has allowed us to create a highly generic service invocation infrastructure that would have been extremely difficult using other Web service frameworks where, often, a client needs to know significantly more about the data schema and service invocation process. Building a framework that focuses on semantics, rather than syntax - not only for the data itself, but also for the messaging infrastructure - means that the client can be largely agnostic so as to how to invoke any service, making the necessary decisions on an *ad hoc* basis at invocation-time. For example, in SADI-Galaxy, when combining different SADI services, the RDF of the intermediate steps can just as easily be consumed by any other RDF-based tool.

Galaxy’s easy-to-use platform for storing data, programs for analysing data, and the resulting workflows is an ideal “ecosystem” within which to provide SADI’s data retrieval and analysis functionalities to our target end-users in a very familiar and straightforward manner. We hope that, by providing SADI services within the widely-used Galaxy platform, we can encourage the more rapid adoption of these powerful new Semantic Web technologies, with SADI-Galaxy acting as the *de facto* interface between these two projects.

## Availability and requirements

-  
**Project name**: SADI-Galaxy.
-  
**Project home page**: http://github.com/mikel-egana-aranguren/SADI-Galaxy^d^.
-  
**Operating system(s)**: UNIX-based (GNU/Linux, Mac OS X, *BSD, *etc.*).
-  
**Programming language**: Java, Python, Shell Script, and Sed.
-  
**Other requirements**: a working Galaxy server (http://galaxyproject.org/).
-  
**License**: General Public License (GPL), version 3.

## Endnotes

^a^ Different “SADI tool-sets” can be added simultaneously to the Galaxy tools menu, by executing the Tool-generator sequentially (with different SPARQL queries) and by editing the title in the Galaxy tools menu each time a new tool set is added (The default title is SADI SERVICES, as shown in Figure 2). This way a researcher can organise SADI services in meaningful groups, having all the groups available in the same Galaxy interface.

^b^ The use cases can be explored and executed in the following Galaxy page (Also available in the http://biordf.org:8983Galaxy server through “shared data” and then “published pages”): http://biordf.org:8983/u/mikel-egana-aranguren/p/sadi-galaxy-jbms-use-cases. In order to reproduce a use case a user must be created in the Galaxy server and the history and the workflow of the use case imported, so that the first item of the history can be used as input for the workflow.

^c^ A locally modified version of SPARQL tools was used, adding the possibility of rendering user-defined namespaces. A patch has been submitted to the original author for inclusion on the Galaxy tool shed repository; the modified version can be obtained at http://github.com/mikel-egana-aranguren/SPARQL_tools_tab2rdf.

^d^ See also TAB2RDF (http://github.com/mikel-egana-aranguren/SPARQL_tools_tab2rdf), SADI generic client (http://toolshed.g2.bx.psu.edu/repos/mikel-egana-aranguren/sadi_generic/) and SPARQL Galaxy (http://toolshed.g2.bx.psu.edu/repos/mikel-egana-aranguren/sparql_galaxy/).

## Abbreviations

RDF: Resource description framework
OWL: Web ontology language
SADI: Semantic automated discovery and integration
SPARQL: SPARQL protocol and RDF query language
TSV: Tab separated values.

## References

[1] World Wide Web Consortium: **World wide web consortium****.** [http://www.w3.org/]. Online; accessed 28-March-2012

[2] W3C: **RDF current status****.** [http://www.w3.org/standards/techs/rdf]. Online; accessed 28-March-2012

[3] W3C: **OWL Web Ontology Language current status****.** [http://www.w3.org/standards/techs/owl]. Online; accessed 28-March-2012

[4] Jain E, Bairoch A, Duvaud S, Phan I, Redaschi N, Suzek B, Martin M, McGarvey P, Gasteiger E (2009). **Infrastructure for the life sciences: design and implementation of the UniProt website**. BMC Bioinformatics.

[5] Jupp S, Malone J, Bolleman J, Brandizi M, Davies M, Garcia L, Gaulton A, Gehant S, Laibe C, Redaschi N, Wimalaratne SM, Martin M, Le Novère N, Parkinson H, Birney E, Jenkinson AM (2014). **The EBI RDF platform: linked open data for the life sciences**. Bioinformatics.

[6] Kosuge T, Mashima J, Kodama Y, Fujisawa T, Kaminuma E, Ogasawara O, Okubo K, Takagi T, Nakamura Y (2013). **DDBJ progress report: a new submission system for leading to a correct annotation**. Nucleic Acids Res.

[7] Belleau F, Nolin M, Tourigny N, Rigault P, Morissette J (2008). **Bio2RDF: Towards a mashup to build bioinformatics knowledge systems**. J Biomed Inform.

[8] Sherry ST, Ward MH, Kholodov M, Baker J, Phan L, Smigielski EM, Sirotkin K (2001). **dbSNP: the NCBI database of genetic variation**. Nucleic Acids Res.

[9] Hamosh A, Scott AF, Amberger JS, Bocchini CA, McKusick VA (2005). **Online Mendelian Inheritance in Man (OMIM): a knowledgebase of human genes and genetic disorders**. Nucleic Acids Res.

[10] Kanehisa M, Goto S (2000). **KEGG: kyoto encyclopedia of genes and genomes**. Nucleic Acids Res.

[11] Maloney C: **RESTful API to NCBI’s Entrez Utilities (E-utilities)****.** [http://pypi.python.org/pypi/eutils]. Online; accessed 26-November-2013

[12] Wilkinson M, Vandervalk B, McCarthy L (2011). **The semantic automated discovery and integration (SADI), web service design-pattern, API and reference implementation**. J Biomed Semantics.

[13] Wolstencroft K, Haines R, Fellows D, Williams A, Withers D, Owen S, Soiland-Reyes S, Dunlop I, Nenadic A, Fisher P, Bhagat J, Belhajjame K, Bacall F, Hardisty A, de la Hidalga AN, Balcazar Vargas MP, Sufi S, Goble C (2013). **The Taverna workflow suite: designing and executing workflows of web services on the desktop, web or in the cloud**. Nucleic Acids Res.

[14] IO Informatics: **IO Informatics Sentient Knowledge Explorer****.** [http://www.io-informatics.com/products/sentient-KE.html]. Online; accessed 26-November-2013

[15] Goecks J, Nekrutenko A, Taylor J, Galaxy Team (2010). **Galaxy: a comprehensive approach for supporting accessible, reproducible, and transparent computational research in the life sciences**. Genome Biol.

[16] Galaxy project: **Galaxy Tool XML File****.** [http://wiki.g2.bx.psu.edu/Admin/Tools/ToolConfigSyntax]. Online; accessed 28-March-2012

[17] Bizer C, Heath T, Berners-Lee T (2009). **Linked data - the story so far**. Int J Semantic Web Inf Syst (IJSWIS).

[18] Aranguren ME: **SADI generic****.** [http://toolshed.g2.bx.psu.edu/repos/mikel-egana-aranguren/sadi_generic/]. Online; accessed 26-November-2013

[19] W3C: **SPARQL current status****.** [http://www.w3.org/standards/techs/sparql]. Online; accessed 28-March-2012

[20] Berman HM, Westbrook J, Feng Z, Gilliland G, Bhat TN, Weissig H, Shindyalov IN, Bourne PE (2000). **The protein data bank**. Nucleic Acids Res.

[21] Sem4j: **SPARQL tools****.** [http://toolshed.g2.bx.psu.edu/repos/sem4j/sparql_tools]. Online; accessed 26-November-2013

[22] Aranguren ME: **SPARQL Galaxy****.** [http://toolshed.g2.bx.psu.edu/repos/mikel-egana-aranguren/sparql_galaxy]. Online; accessed 26-November-2013

[23] SADI: **UniProt record SADI input OWL class****.** [http://purl.oclc.org/SADI/LSRN/UniProt_Record]. Online; accessed 28-March-2012

[24] Kasprzyk A (2011). **BioMart: driving a paradigm change in biological data management**. Database.

[25] Dhamanaskar A, Cotterell ME, Zheng JZ, Miller JA, Kissinger JC, Stoeckert CJ (2012). **Suggestions in galaxy workflow design based on ontologically annotated services**. Proceedings of the 7th International Conference on Formal Ontology in Information Systems (FOIS’12).

[26] Wang R, Brewer D, Shastri S, Swayampakula S, Miller JA, Kraemer ET, Kissinger JC (2009). **Adapting the galaxy bioinformatics tool to support semantic web service composition**. Proceedings of the 2009 Congress on Services - Part I.

[27] Abouelhoda M, Issa S, Ghanem M (2012). **Tavaxy: Integrating taverna and galaxy workflows with cloud computing support**. BMC Bioinformatics.

